# MicroRNA Processing Pathway-Based Polygenic Score for Clear Cell Renal Cell Carcinoma in the Volga-Ural Region Populations of Eurasian Continent

**DOI:** 10.3390/genes13071281

**Published:** 2022-07-20

**Authors:** Elizaveta Ivanova, Irina Gilyazova, Valentin Pavlov, Adel Izmailov, Galiya Gimalova, Alexandra Karunas, Inga Prokopenko, Elza Khusnutdinova

**Affiliations:** 1 Institute of Biochemistry and Genetics—Subdivision of the Ufa Federal Research Centre of the Russian Academy of Sciences, 450054 Ufa, Russia; lizavetaivanova91@gmail.com (E.I.); galiyagimalova@gmail.com (G.G.); carunas@list.ru (A.K.); elzakh@mail.ru (E.K.); 2 Bashkir State Medical University, 450008 Ufa, Russia; vpavlov3@yandex.ru (V.P.); izmailov75@mail.ru (A.I.); 3 Department of Clinical & Experimental Medicine, University of Surrey, Guildford GU2 7XH, UK; i.prokopenko@surrey.ac.uk; 4 UMR 8199—EGID, Institut Pasteur de Lille, CNRS, University of Lille, F-59000 Lille, France

**Keywords:** renal cell carcinoma, genetic risk score

## Abstract

The polygenic scores (PGSs) are developed to help clinicians in distinguishing individuals at high risk of developing disease outcomes from the general population. Clear cell renal cell carcinoma (ccRCC) is a complex disorder that involves numerous biological pathways, one of the most important of which is responsible for the microRNA biogenesis machinery. Here, we defined the biological-pathway-specific PGS in a case-control study of ccRCC in the Volga-Ural region of the Eurasia continent. We evaluated 28 DNA SNP variants, located in microRNA biogenesis genes, in 464 individuals with clinically diagnosed ccRCC and 1042 individuals without the disease. Individual genetic risks were defined using the SNP-variant effects derived from the ccRCC association analysis. The final weighted and unweighted PGS models were based on 21 SNPs, and 7 SNPs were excluded due to high LD. In our dataset, microRNA-machinery-weighted PGS revealed 1.69-fold higher odds (95% CI [1.51–1.91]) for ccRCC risk in individuals with ccRCC compared with controls with a *p*-value of 2.0 × 10^−16^. The microRNA biogenesis pathway weighted PGS predicted the risk of ccRCC with an area under the curve (AUC) = 0.642 (95%nCI [0.61–0.67]). Our findings indicate that DNA variants of microRNA machinery genes modulate the risk of ccRCC in Volga-Ural populations. Moreover, larger powerful genome-wide association studies are needed to reveal a wider range of genetic variants affecting microRNA processing. Biological-pathway-based PGSs will advance the development of innovative screening systems for future stratified medicine approaches in ccRCC.

## 1. Introduction

Renal cell carcinoma (RCC) is a malignancy of the kidney, accounting for approximately 90% of all kidney cancers. It is the tenth most common form of cancer in the world [1]. According to statistical data, the incidence of renal cancer is steadily increasing [2], with more than 179,000 deaths from renal cell cancer registered solely in 2020 [3].

The major histologic subtype of RCC is clear cell RCC (ccRCC), accounting for ~75–80% [4]. CcRCC is highly aggressive, with approximately 30% of individuals showing metastasis at the time of diagnosis and poor prognosis. The issue of obtaining better knowledge about ccRCC predisposition is related to a number of clinical and research areas such as screening, risk stratification, diagnostics, disease severity, prognosis, and clinical trials of new drugs. The identification of specific genetic markers affecting the risk of ccRCC involves clarifying the diagnosis, adjusting the methods of treatment, and developing better prognosis approaches and tools that are sensitive enough for early identification of ccRCC pathogenesis. Recent studies demonstrated the potential of PGS as a useful instrument in determining the risk of diverse diseases, including various cancers [5,6,7]. PGSs combine the effects of disease-associated single-nucleotide polymorphisms (SNPs), providing marked cancer risk stratification in the general population [8].

MicroRNAs play an important role in ccRCC [9,10,11,12,13]. MicroRNAs (miRNAs) are a very important part of the post-transcriptional mechanism of gene expression and are involved in carcinogenesis. SNPs in genes of microRNA biogenesis pathways can affect mature microRNA levels and lead to the modulation of a wide range of processes regulated by them [14].

Because miRNAs cover a wide range of regulated genes, SNPs in miRNA genes and target sites can function as modifiers of the effects of microRNA on phenotypes and disease susceptibility [15]. Moreover, SNPs located in biogenesis and miRNA precursor genes can have complex effects, influencing miRNA maturation, functional strand selection, and target mRNA definition. The presence of SNPs either in the genomic miRNA sequences or in the 3′UTR of cancer-associated genes can influence miRNA-dependent regulation, thus changing tumor susceptibility [16].

The identification of germline genetic variants that predict ccRCC risk and that may serve as additional markers to somatic alterations is a promising approach. Although genetic polymorphisms of miRNA biogenesis machinery genes are widely implicated in cancer development [17,18], polygenic risk scales involving miRNA biogenesis genes have not been previously evaluated. The current literature on PGS in ccRCC is scarce compared with the vast number of reported SNPs associated with the development of the disease. Most models have been constructed in individuals of European origin, and ethnic-specific PGS is required to account for genetic variations in different populations.

Here, we evaluated the effects of 28 SNPs located in miRNA machinery genes on ccRCC risk in three ethnic groups living in the Volga-Ural region and implemented a polygenic risk score (PGS) approach to evaluate the cumulative effects of these DNA variants.

## 2. Materials and Methods

### 2.1. Study Sample

All genotyped individuals with ccRCC and controls without the disease were residents of the Volga-Ural region of Eurasia. The study was performed according to the ethical standards of the Bioethics Committee who developed the Declaration of Helsinki of the World Association which governs “the ethical principles of medical research involving human subjects” [19] and with the ethical standards of the Research Ethics Committee of the Institute of Biochemistry and Genetics, a subdivision of the Ufa Federal Research Centre of the Russian Academy of Sciences. Informed written consent was obtained from each participant included in this study. Overall, 464 individuals with nonfamilial ccRCC (cases) were included in the study. All individuals with ccRCC underwent radical nephrectomy at Bashkir State Medical University Clinic. The inclusion criterion for the study was histologically confirmed ccRCC, and such individuals were not prescribed chemotherapy or radiotherapy prior to the collections of blood samples. There were no age, sex, ethnicity, or cancer stage restrictions for participation in the study. The control group comprised 1042 unrelated individuals from the general population residing in the same region and without oncological diseases in their family history, and corresponded to the group of cases in terms of clinical and demographic characteristics.

### 2.2. Blood Sample Collection, DNA Extraction, and Genotyping Procedures

The selection of 28 SNPs in microRNA biogenesis pathway genes and pre-miRNAs for genotyping in study samples was performed using the databases of the International HapMap Project, dbSNP, and miRBase registry and Ensembl [20,21,22,23]. All SNPs have a reported minor allele frequency (MAF) of >0.01 in Europeans.

Peripheral blood samples were collected from each subject and placed into 7 mL vacutainer tubes containing EDTA. Genomic DNA was extracted from blood leukocytes by using conventional proteinase K digestion and phenol-chloroform extraction methods. DNA concentration was measured with a NanoDrop 1000 spectrophotometer (Thermo Fisher Scientific, Fitchburg, WI, USA). Genomic DNA samples were normalized at 50 ng/μL. Genotyping of SNP variants of biogenesis genes and miRNA precursors was carried out using OpenArray technology on a Quant Studio™12K Flex Real-Time PCR System (Applied Biosystems, CA, USA). Samples were analyzed on special slide chips containing a total of 3072 through holes for individual reactions at 33 ηL. A 3 μL reaction mixture (TaqMan^®^ OpenArray^®^ Genotyping MasterMix, Applied Biosystems, Foster City, CA, USA) was mixed with 3 μL of DNA samples in a 384-well plate and then loaded into a custom-designed OpenArray plate preloaded with the genotyping primers and probe for the selected SNPs by using a QuantStudio 12 K Flex AccuFill system. PCR was performed under the following conditions: initial denaturation at 95 °C for 10 min; denaturation at 92 °C for 15 s, and annealing/elongation at 60 °C for 1 min (40 cycles). The genotyping data analysis was performed using the software package TaqMan Genotyper Software v.1.3.

### 2.3. Quality Control (QC) and Association Analysis

For quality control purposes, we evaluated the Hardy–Weinberg equilibrium by comparing the genotype distribution for each SNP variant genotyped with the expectation calculated from the allele frequencies. Overall, the average call rate for all SNPs was 98%. All calculations were performed using *PLINK* 1.9 [24] and the *R* 4.1.1 software tool and environment [25]. For each SNP, allelic ORs for ccRCC with 95% confidence intervals were calculated using logistic regression and assuming a log-additive genetic model. First, the individual genetic risks were determined using PGS based on 28 SNPs located in miRNA biogenesis genes (Table S2 in Supplementary File_3.docx). Further sensitivity analysis was performed and SNPs with r2 >0.2 were excluded due to high LD (Supplementary Materials File_4.docx). Final weighted and unweighted PGS models were based on 21 SNPs (Table 2). Given the unavailability of a training sample from the genome-wide association studies (GWAS) data, an analysis of the logistic regression model was carried out, and the values of the effects (OR, odds ratio) of the studied polymorphic variants were obtained (Table 2). Statistical significance for PGS was defined as *p* < 0.05. We also applied the Bonferroni correction method to the single-variant association analysis for multiple tests, for a total of 21 independent tests (P_Bonferroni_ < 0.05/21), providing us with the study-wide threshold of P_Bonferroni_ < 0.0023.

### 2.4. PGS Calculation

The PGS was calculated for each individual using the risk allele effect weighted sum of all studied polymorphic loci. The risk allele count for each SNP was weighted by its effect size based on its association with ccRCC under a log-additive genetic model implemented within a logistic regression analysis with adjustments for age, sex, and ethnicity. We used *PLINK* 1.9 [24] for logistic regression analysis. For variants with OR <1, we used the inverse OR and 1 (the reported effect allele frequency) so that the association was in the direction of ccRCC risk for all SNPs. Thus, the effect size obtained for our data was used as weights. Additionally, the impact of unweighted PGS on the ccRCC was assessed for sensitivity purposes. As a result, two models of polygenic risk, weighted and unweighted, were compared.

### 2.5. Receiver Operating Characteristic (ROC) Analysis

The distribution of the individual PGSs was compared between the cases and controls, and their discriminatory capacities were evaluated using receiver operating characteristic (ROC) curves in the *R* environment with the *pROC* package [26]. The strength of model to predict ccRCC against controls was assessed by comparing the area under the curve (AUC) of the respective ROC curves, which compares the true-positive rate against the false-positive rate.

## 3. Results

### 3.1. Association Analysis

We used a ccRCC study from the Volga-Ural region, including 464 nonfamilial ccRCC cases and 1042 unrelated population-based controls without cancer, which are described in Table 1.

The analysis of 21 SNPs, located in miRNA biogenesis genes, highlighted the strongest association for rs1057035 in *DICER1* (OR [95% CI] = 1.85 [1.58–2.18], *p*-value = 4.05 × 10^−14^, Table 2). After Bonferroni correction for multiple testing, three SNPs showed significant effect on ccRCC risk in our study (Table 2). These included rs11060845 at *PIWIL1*, rs3809142 at *RAN* gene, and rs1057035 in *DICER1.* Moreover, five additional variants reached nominal significance within ccRCC association analysis, namely, rs595055 at *AGO1*, rs13078 in *DICER1*, rs6505162 at *NSRP1,* rs1991401 at *DDX5,* and rs720012 in *DGCR8*.

**Table 2:** Association between reduced set of 21 DNA variants in miRNA biogenesis genes and ccRCC in Volga-Ural populations.

Gene Name	Chromosome	Position, (GRCh37)	rsID	Risk Allele/Non-Risk Allele	Effect Allele Frequency Cases/Controls	OR (95% CI)	*p*-Value
*AGO1*	1	36,380,133	rs595055	T/C	0.26/0.30	1.20 (0.71–0.99)	0.04
*DDX20*	1	112,308,953	rs197412	T/C	0.47/0.49	1.11 (0.77–1.06)	0.21
*DROSHA*	5	31,435,627	rs4867329	A/C	0.46/0.49	1.11 (0.77–1.05)	0.19
*C5orf22*	5	31,532,789	rs17409893	A/G	0.31/0.32	1.08 (0.78–1.10)	0.37
*XPO5*	6	43,492,578	rs2257082	G/A	0.33/0.33	1.00 (0.84–1.18)	0.98
*AGO2*	8	141,555,862	rs3864659	A/C	0.12/0.14	1.15 (0.69–1.10)	0.24
*AGO2*	8	141,594,460	rs7005286	T/C	0.23/0.21	1.11 (0.93–1.33)	0.26
*MIR196A2*	12	54,385,599	rs11614913	T/C	0.38/0.37	1.06 (0.91–1.25)	0.45
*PIWIL1*	12	130,852,174	rs11060845	G/T	0.07/0.11	**1.69 (0.44–0.79)**	**4.32 × 10^−4^**
*RAN*	12	131,355,546	rs3809142	C/T	0.11/0.16	**1.57 (0.50–0.81)**	**3.05 × 10^−4^**
*DICER1*	14	95,554,142	rs1057035	C/T	0.44/0.29	**1.85 (1.58-2.18)**	**4.05 × 10^−14^**
*DICER1*	14	95,556,747	rs13078	T/A	0.13/0.17	1.28 (0.62–0.98)	0.03
*GEMIN4*	17	649,232	rs3744741	C/T	0.15/0.18	1.19 (0.68–1.04)	0.11
*GEMIN4*	17	649,505	rs4968104	T/A	0.21/0.22	1.06 (0.78–1.14)	0.55
*GEMIN4*	17	649,935	rs2740348	G/C	0.19/0.17	1.20 (0.98–1.47)	0.07
microRNA-423 (*NSRP1*)	17	28,444,183	rs6505162	A/C	0.50/0.46	1.17 (1.00–1.37)	0.04
*DDX5*	17	62,502,435	rs1991401	G/A	0.44/0.38	1.25 (1.07–1.47)	4.99 × 10^−3^
*MIR27A*	19	13,947,292	rs895819	T/C	0.34/0.35	1.06 (0.80–1.11)	0.45
*DGCR8*	22	20,098,544	rs417309	G/A	0.08/0.10	1.17 (0.65–1.12)	0.26
*DGCR8*	22	20,098,582	rs720012	A/G	0.24/0.20	1.24 (1.03–1.49)	0.02
*DGCR8*	22	20,098,882	rs720014	C/T	0.24/0.21	1.15 (0.95–1.40)	0.14

Abbreviations: OR—odds ratio; CI—confidence interval. Association test statistics for three variants surviving the multiple testing correction (see **Methods**) is highlighted in bold characters.

As a result of weighted PGS analysis, 1.69 times higher chances (95% CI [1.51–1.91]) of the ccRCC risk were revealed in cases compared with controls, with a statistically significant *p*-value of 2.0 × 10^−16^. Unweighted PGS models showed a lower odds ratio OR (95% CI) of 1.60 (1.42–1.80) with a less significant *p*-value of 2.95 × 10^−14^ (Supplementary Materials File_1.docx).

### 3.2. Receiver Operator Characteristic Analysis

**Figure 1:**
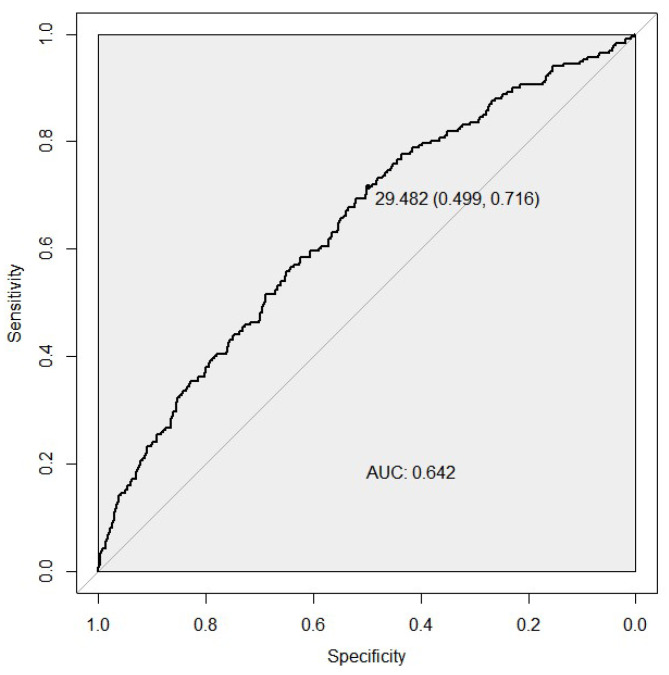
ROC curves assessing the discriminative power of the weighted PGS model for the ccRCC risk. The best predictive point is shown with the ideal cut-off for the PGS and with estimates for specificity and sensitivity at that point. AUC, area under the curve.

We compared the discriminative ability of two PGS models for the case/control status by developing the ROC curves. The microRNA biogenesis pathway weighted PGS predicted the risk of developing ccRCC with an AUC of 0.642 (95% CI [0.61–0.67], sensitivity of 0.71, and a specificity of 0.50 (Figure 1). For the unweighted PGS model, see Figure S1 in Supplementary File_1.docx.

## 4. Discussion

In this study, we evaluated the ability of DNA variants to predict the risk of ccRCC in microRNA biogenesis pathway loci. We found suggestive evidence of the combined effects of the weighted PGS on ccRCC risks in the Volga-Ural region of Eurasia. We included 28 SNPs of microRNA biogenesis pathway genes that recently showed an association with the development of diverse types of cancers, including clear cell carcinoma. As a result, we found the combination of 21 microRNA biogenesis SNPs could predict 1.69 times higher chances (95% CI [1.51–1.91]) of ccRCC. We also evaluated the PheWAS data of the studied SNPs and found a correlation with several traits including urological cancers, in particular bladder cancer and other types of malignances (Supplementary File_2.docx).

Recently, it was described that a gene pathway-based PGS approach for genetic risk prediction for human phenotypes may shed light on disease biology and identify core gene networks that contribute the most risk to a polygenic disorder [27]. Today, the most popular procedure used to investigate genetic variants is GWAS, which allows for the simultaneous analysis of millions of DNA variants. As a result of this approach, we can identify hundreds of disease susceptibility loci containing low-risk variants, and only a few of them will be the most significantly associated with disease risk. At the same time, in RCC, with population prevalence of 4.91/100,000 [3], the assessment of the influence of certain genotypes and alleles was traditionally most often carried out by identifying the association of individual polymorphisms with the risk of developing the disease.

Direct evidence of the inherited predisposition to RCC is provided by a number of rare cancer syndromes with defined germline mutations in 11 genes (*BAP1*, *FLCN*, *FH*, *MET*, *PTEN*, *SDHB*, *SDHC*, *SDHD*, *TSC1*, *TSC2*, and *VHL*), associated with the development of different RCC subtypes [28]. Nevertheless, all of these genes can explain only a two-fold increased risk of RCC in first-degree relatives of individuals with RCC [29]. According to estimates of previous GWAS, the established RCC risk loci account for only about 10% of the familial risk of disease [30].

Growing evidence suggests that SNPs in core components of miRNA biogenesis may impair or enhance miRNA processing efficiency or function, which can function as an oncogene or tumor suppressor [31]. Evidence from published reports highlights that SNPs in miRNAs, which encode their biogenesis pathway and target binding sites, may affect the regulatory capacity of miRNAs by affecting miRNA processing or miRNA–mRNA interactions [32]. To date, most of the studies in this field have had a case-control design and have been based on a candidate gene approach [15]. For instance, in one study of renal cancer 41 SNPs in 11 miRNA biogenesis genes were analyzed [33]. Two SNPs in the *GEMIN4* gene were significantly associated with the renal carcinoma risk. Moreover, the common *GEMIN4* H3 haplotype (wmmwww, where w is the wild-type allele, and m is the minor allele), consisting of six nonsynonymous SNPs in the order rs910924, rs2740348, rs7813, rs3744741, rs1062923, and rs4968104, was protective of developing RCC (OR = 0.66, 95% CI: 0.45–0.97) in the respective haplotype analysis [33].

The number of studies using PGS in RCC is rather limited. Thus, in one study, PGS analysis of 13 GWAS-established ccRCC SNPs was recently performed for tumor molecular subtypes: ccA, characterized by overexpression of genes associated with hypoxia, angiogenesis, and fatty and organic acid metabolism; and the poorer-prognosis ccB, overexpressing genes regulating epithelial-to-mesenchymal transition, the cell cycle, and wound healing. This GWAS-based PGS signal was associated with both ccA and, in particular, ccB tumors (90th vs. 10th percentile: OR (95% CI) = 1.82 (1.11–2.99), *p*-value = 0.02 and OR (95% CI) = 2.87 (1.64–5.01), *p*-value = 2 × 10^−4^, respectively) [34]. In addition, the genetic risk score based on leukocyte telomere-length-associated SNPs was connected with the risk of recurrence in individuals with renal cell carcinoma [35]. Another study showed a relatively low AUC (95% CI) of 0.567 (0.54–0.59) for PGS of the 15 genetic variants identified by previous GWAS in association with the risk of renal cancer [36].

## 5. Conclusions

Despite the investigation of molecular-pathway-based SNPs allowing for a determination of significant associations with RCC, GWAS is urgently needed to discover new uncommon and rare variants that explain a vital group of the variation in complex characteristics.

## Figures and Tables

**Table 1:** Characteristics of study population.

Characteristic	Individuals with ccRCC	Controls
Total	464	1042
Sex, n (%)		
Male	282 (60.8)	519 (49.8)
Female	182 (39.2)	523 (50.2)
Age, years, mean ± SD	56.01±0.71	53.6 ± 0.66
TNM stage		
I-II, n (%)	267 (57.5)	–
III-IV, n (%)	197 (42.5)	–
Ethnicity, n (%)		
Bashkir	78 (16.8)	142 (13.6)
Tatar	174 (37.5)	457 (43.9)
Russian	212 (45.7)	443 (42.5)

## Supplementary Materials

The following supporting information can be downloaded at: https://www.mdpi.com/article/10.3390/genes13071281/s1, (Supplementary File_1) Figure S1: ROC curves assessing the discriminative power of the weighted PRS model for the ccRCC risk; (Supplementary File_2) Table S1: PheWAS data of studied SNPs in microRNA biogenesis pathway genes; (Supplementary File_3) Table S2: Association between 28 DNA variants in miRNA biogenesis genes and ccRCC in Volga-Ural populations; Figures S2 and S3. ROC curves assessing the discriminative power of the unweighted PRS model for the ccRCC risk; (Supplementary Materials File_4) Table S3: Linkage disequilibium using the European+Asian populations LD estimates from the 1000 Genomes Project (www.ldlink.nci.nih.gov) between SNPs located within 500 kb from each other.
