# Supplementary material for: MicroRNA Processing Pathway-Based Polygenic Score for Clear Cell Renal Cell Carcinoma in the Volga-Ural Region Populations of Eurasian Continent
Source: Genes (Basel). 2022 Jul 20;13(7):1281. doi: 10.3390/genes13071281 (PMC9324265; doi:10.3390/genes13071281)
Supplement: Supplementary file 1 [file genes-13-01281-s001.zip › Supplementary File_1.pdf]

Unweighted PRS analysis demonstrated OR(95%CI)=1.60(1.42-1.80) of the ccRCC risk with a statistical significance P-value=2.95×10^{-14}. The microRNA biogenesis pathway unweighted PRS predicted the risk of developing ccRCC with AUC=0.629 (95%CI[0.60–0.66], sensitivity of 0.44 and a specificity of 0.76 (Figure S1).

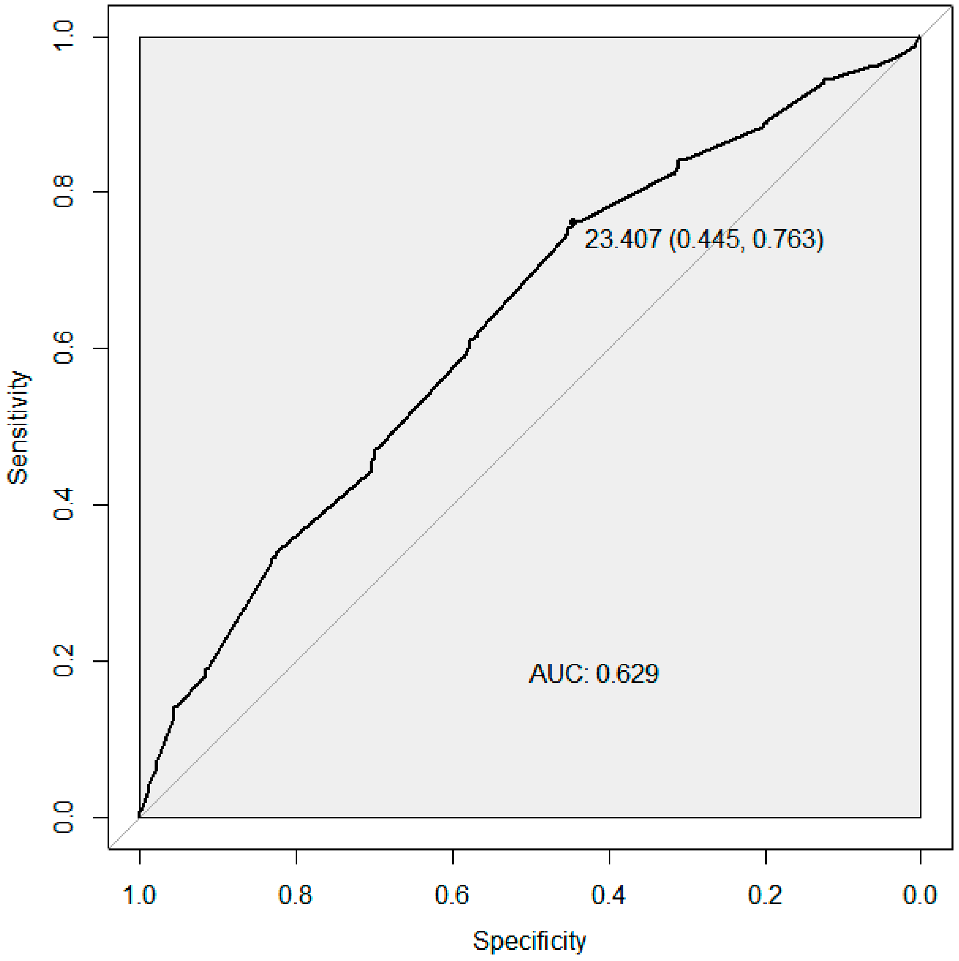

Figure S1. ROC curves assessing the discriminative power of the weighted PRS model for the ccRCC risk. The best predictive point is shown with the ideal cut-off for the PRS and with estimates for specificity and sensitivity at that point. AUC, area under the curve.
