# Supplementary material for: MicroRNA Processing Pathway-Based Polygenic Score for Clear Cell Renal Cell Carcinoma in the Volga-Ural Region Populations of Eurasian Continent
Source: Genes (Basel). 2022 Jul 20;13(7):1281. doi: 10.3390/genes13071281 (PMC9324265; doi:10.3390/genes13071281)
Supplement: Supplementary file 1 [file genes-13-01281-s001.zip › Supplementary File_2.pdf]

Table S1. PheWAS data of studied SNPs in microRNA biogenesis pathway genes

Feature	Chr	RSID	Gene	Allele	Position	Phenotype	β	SE(β)	p-value
Urological cancers	1	rs595055	AGO1	T	36380133	Malignant neoplasm of bladder	-0.13	0.05	6.35x10^-3
						Cancer of bladder	-0.11	0.04	9.50x10^-3
	17	rs2740348	GEMIN4	G	649935	Cancer of bladder	0.07	0.04	0.06
	17	rs2740351	FAM57A (GEMIN4)	G	643426	Malignant neoplasm of other urinary organs	0.25	0.12	0.04
	17	rs7813	GEMIN4	G	648186	Malignant neoplasm of other urinary organs	0.26	0.13	0.04
Other malignant neoplasms with the most significant p-value	1	rs197412	DDX20	C	112308953	Cancer of brain and nervous system	0.18	0.06	3.96x10^-3
						Benign neoplasm of other female genital organs Secondary malignant neoplasm	0.27	0.10	6.60x10^-3
						Secondary malignant neoplasm	-0.04	0.01	9.37x10^-3
	1	rs11584657	FAM212B	T	112296374	Secondary malignant neoplasm	-0.06	0.02	6.30x10^-3
	5	rs4867329	DROSHA	C	31435627	Bone cancer	0.39	0.13	2.34x10^-3
						Multiple myeloma	0.18	0.06	3.43x10^-3
						Benign neoplasm of other endocrine glands and related structures	-0.13	0.05	6.56x10^-3
	6	rs2257082	XPO5	A	43492578	Malignant neoplasms of ovary	0.12	0.04	3.82x10^-4
						Malignant neoplasm of ovary and other uterine adnexa	0.12	0.04	4.66x10^-4
						Cervical cancer	0.12	0.04	2.54x10^-3
						Cervical cancer and dysplasia	0.08	0.03	4.14x10^-3
						Cancer of other female genital organs	0.09	0.03	7.32x10^-3
	8	rs3864659	AGO2	C	141555862	Cancer of other endocrine glands	-0.61	0.23	8.82x10^-3
	22	rs417309	DGCR8	A	20098544	Malignant neoplasm of rectum, rectosigmoid junction, and anus	0.18	0.05	1.01x10^-3
						Second malignancy of brain/spine	0.23	0.09	7.57x10^-3
