# Supplementary material for: MicroRNA Processing Pathway-Based Polygenic Score for Clear Cell Renal Cell Carcinoma in the Volga-Ural Region Populations of Eurasian Continent
Source: Genes (Basel). 2022 Jul 20;13(7):1281. doi: 10.3390/genes13071281 (PMC9324265; doi:10.3390/genes13071281)
Supplement: Supplementary file 1 [file genes-13-01281-s001.zip › Supplementary File_3.pdf]

Table S2. Association between 28 DNA variants in miRNA biogenesis genes and ccRCC in Volga-Ural populations.

Gene name	Chromosome	Position, (GRCh37)	rsID	Risk Allele/ Non-risk allele	Effect allele frequency cases/controls	OR (95%CI)	P-value
AGO1	1	36,380,133	rs595055	T/C	0.26/0.30	**1.20 (0.71-0.99)**	**0.04**
FAM212B	1	112,296,374	rs11584657	C/T	0.20/0.20	1.02 (0.80-1.19)	0.82
DDX20	1	112,308,953	rs197412	T/C	0.47/0.49	1.11 (0.77-1.06)	0.21
DROSHA	5	31,401,003	rs642321	T/C	0.26/0.25	1.05 (0.88-1.26)	0.57
DROSHA	5	31,401,447	rs10719	A/G	0.31/0.30	1.06 (0.90-1.25)	0.51
DROSHA	5	31,435,627	rs4867329	A/C	0.46/0.49	1.11 (0.77-1.05)	0.19
C5orf22	5	31,532,789	rs17409893	A/G	0.31/0.32	1.08 (0.78-1.10)	0.37
XPO5	6	43,492,578	rs2257082	G/A	0.33/0.33	1.00 (0.84-1.18)	0.98
AGO2	8	141,555,862	rs3864659	A/C	0.12/0.14	1.15 (0.69-1.10)	0.24
AGO2	8	141,594,460	rs7005286	T/C	0.23/0.21	1.11 (0.93-1.33)	0.26
MIR196A2	12	54,385,599	rs11614913	T/C	0.38/0.37	1.06 (0.91-1.25)	0.45
PIWIL1	12	130,852,174	rs11060845	G/T	0.07/0.11	**1.69 (0.44-0.79)**	**$4.32{\times}10^{-4}$**
PIWIL1	12	130,856,316	rs10773771	T/C	0.40/0.43	1.14 (0.74-1.03)	0.11
RAN	12	131,355,546	rs3809142	C/T	0.11/0.16	**1.57 (0.50-0.81)**	**$3.05{\times}10^{-4}$**
DICER1	14	95,554,142	rs1057035	C/T	0.44/0.29	**1.85 (1.58-2.18)**	**$4.05{\times}10^{-14}$**
DICER1	14	95,556,747	rs13078	T/A	0.13/0.17	**1.28 (0.62-0.98)**	**0.03**
FAM57A (GEMIN4)	17	643,426	rs2740351	G/A	0.41/0.40	1.06 (0.91-1.24)	0.47
GEMIN4	17	648,186	rs7813	G/A	0.41/0.39	1.04 (0.89-1.22)	0.59
GEMIN4	17	649,232	rs3744741	C/T	0.15/0.18	1.19 (0.68-1.04)	0.11
GEMIN4	17	649,505	rs4968104	T/A	0.21/0.22	1.06 (0.78-1.14)	0.55
GEMIN4	17	649,935	rs2740348	G/C	0.19/0.17	1.20 (0.98-1.47)	0.07
microRNA-423 (*NSRP1*)	17	28,444,183	rs6505162	A/C	0.50/0.46	**1.17 (1.00-1.37)**	**0.04**
DDX5	17	62,502,435	rs1991401	G/A	0.44/0.38	**1.25 (1.07-1.47)**	**$4.99{\times}10^{-3}$**
MIR27A	19	13,947,292	rs895819	T/C	0.34/0.35	1.06 (0.80-1.11)	0.45
*DGCR8**	22	20,098,359	rs1640299	G/T	0.39/0.38	1.06 (0.90-1.25)	0.51
*DGCR8**	22	20,098,544	rs417309	G/A	0.08/0.10	1.17 (0.65-1.12)	0.26
*DGCR8**	22	20,098,582	rs720012	A/G	0.24/0.20	**1.24 (1.03-1.49)**	**0.02**
*DGCR8**	22	20,098,882	rs720014	C/T	0.24/0.21	1.15 (0.95-1.40)	0.14

Weighted PRS analysis demonstrated OR(95%CI)=1.60(1.42-1.80) of the ccRCC risk with a statistical significance P-value=4.20×10^{-15}. The microRNA biogenesis pathway weighted PRS predicted the risk of developing ccRCC with AUC=0.637 (95%CI[0.61–0.67], sensitivity of 0.58 and a specificity of 0.66 (Figure S2).

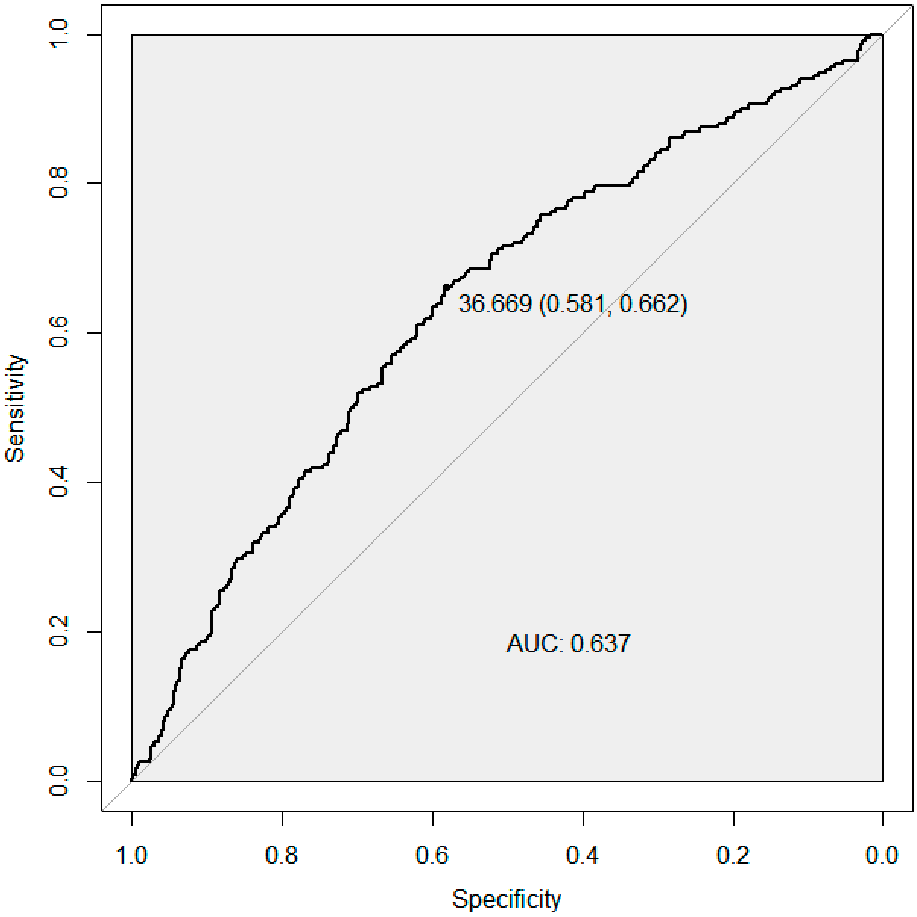

Figure S2. *ROC curves assessing the discriminative power of the weighted PRS model for the ccRCC risk. The best predictive point is shown with the ideal cut-off for the PRS and with estimates for specificity and sensitivity at that point. AUC, area under the curve.*

Unweighted PRS analysis demonstrated OR(95%CI)=1.50(1.34-1.69) of the ccRCC risk with a statistical significance P-value=4.15×10^{-12}. The microRNA biogenesis pathway unweighted PRS predicted the risk of developing ccRCC with AUC=0.622 (95%CI[0.59–0.65], sensitivity of 0.55 and a specificity of 0.69 (Figure S3).

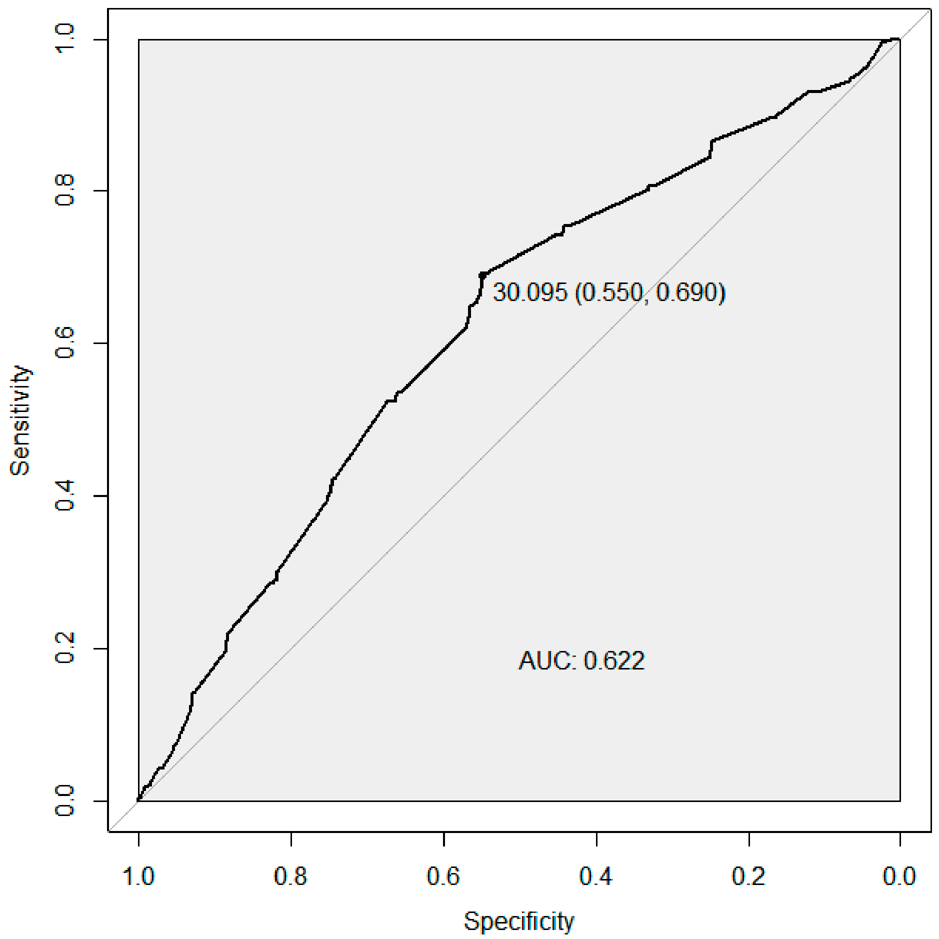

Figure S3. *ROC curves assessing the discriminative power of the unweighted PRS model for the ccRCC risk. The best predictive point is shown with the ideal cut-off for the PRS and with estimates for specificity and sensitivity at that point. AUC, area under the curve.*
