# Supplementary material for: MicroRNA Processing Pathway-Based Polygenic Score for Clear Cell Renal Cell Carcinoma in the Volga-Ural Region Populations of Eurasian Continent
Source: Genes (Basel). 2022 Jul 20;13(7):1281. doi: 10.3390/genes13071281 (PMC9324265; doi:10.3390/genes13071281)
Supplement: Supplementary file 1 [file genes-13-01281-s001.zip › Supplementary Materials_File 4.pdf]

Table S3. Linkage disequilibium using the European+Asian populations LD estimates from the 1000 Genomes Project (www.ldlink.nci.nih.gov) between SNPs located within 500kb from each other

SNP pair	r^2
rs11584657-rs197412	0.309
rs642321-rs10719	0.649
rs4867329-rs10719	0.338
rs4867329-rs17409893	0.029
rs3864659-rs7005286	0.007
rs11060845-rs10773771	0.114
rs1057035-rs13078	0.007
rs2740351-rs7813	0.974
rs2740351- rs3744741	0.171
rs2740351- rs4968104	0.452
rs2740351-rs2740348	0.264
rs7813-rs3744741	0.173
rs7813- rs4968104	0.455
rs7813- rs2740348	0.264
rs3744741-rs4968104	0.079
rs3744741-rs2740348	0.046
rs4968104-rs2740348	0.036
rs1640299-rs417309	0.041
rs1640299-rs720012	0.262
rs1640299-rs720014	0.364
rs417309-rs720012	0.026
rs417309-rs720014	0.015
rs720012-rs720014	0.095
